# Allergen sensitization patterns: Allergic rhinitis with multimorbidity versus alone—A real‐world study

**DOI:** 10.1002/clt2.70030

**Published:** 2025-01-22

**Authors:** Ya‐Ting Li, Qi‐Qing Ye, Ya‐Xin Lu, Ke‐Xin Yang, Ping‐Ping Zhang, Chang Chen, Min Zhou, Pei‐Ying Feng, Zhuang‐Gui Chen

**Affiliations:** ^1^ Department of Allergy Third Affiliated Hospital of Sun Yat‐sen University Guangzhou China; ^2^ Children's Medicine Centre Third Affiliated Hospital of Sun Yat‐sen University Guangzhou China; ^3^ Clinical Data Center The Third Affiliated Hospital of Sun Yat‐sen University Guangzhou China; ^4^ Zhongshan School of Medicine Sun Yat‐sen University Guangzhou China; ^5^ Department of Dermatology The Third Affiliated Hospital of Sun Yat‐sen University Guangzhou China

**Keywords:** allergen sensitization patterns, allergic multimorbidity, allergic rhinitis, polysensitization, specific immunoglobulin E

## Abstract

**Background:**

Allergic rhinitis (AR) multimorbidity may need to be considered a specific disease because of distinct clinical and immunological differences from AR alone. Allergic multimorbidity often involves polysensitization, where allergen‐specific immunoglobulin E (IgE) plays a significant role.

**Objective:**

This study aims to explore differences in allergen IgE sensitization patterns between AR alone and AR multimorbidity.

**Methods:**

A real‐world case‐control study was conducted with patients diagnosed with AR. Multivariate logistic regression analyzed the associations between AR multimorbidity and allergen sensitivity, allergen‐specific IgE levels, and the count of positive allergens.

**Results:**

A cohort of 2275 patients with AR was included, of which 1100 (48.4%) presented with AR alone, while 1175 (51.6%) exhibited AR multimorbidity. Patients with AR multimorbidity had a more diverse allergen profile than those with AR alone. An increased number of positive ingested allergens had a higher odds ratio (OR) for AR multimorbidity compared with inhaled allergens (1.46 vs. 1.96) across all phenotypes. Sensitization to allergens and their allergen‐specific IgE levels, including dust mites, cat dander, and milk (*p* < 0.05), were associated with AR multimorbidity. In children, cat and dog dander were significant allergens associated with AR multimorbidity (*p* < 0.05).

**Conclusions:**

Allergen sensitization patterns in AR multimorbidity differ from those in AR alone. Polysensitization, particularly to ingested allergens, increases the risk of allergic multimorbidity. The risk of allergic multimorbidity also increases with specific allergen positivity and higher allergen‐specific IgE levels.

## 1 INTRODUCTION

Allergic rhinitis (AR) has become a common public health problem worldwide with about 400 million patients and its incidence is on the rise, which is also prone to be combined with a variety of other allergic diseases, called AR multimorbidity.^1^

The MeDALL (Mechanisms of the Development of Allergy) suggests that allergic diseases are intricately shaped by both genetic and environmental determinants, and since there are important clinical and immunological differences in mono‐ and polysensitized subjects, potentially categorizing them into distinct allergic phenotypes.^2^ Research suggests that allergic multimorbidity should be considered a discrete clinical entity, supported by genetic investigations.^3^, ^4^, ^5^ The EuroPrevall‐iFAAM birth cohort showed that the actual prevalence of triple multimorbidity (AR + asthma [AS]+ atopic dermatitis [AD]) was much higher than the theoretical prevalence (1.3% vs. 0.13%).^6^ AR multimorbidity is positively associated with the frequency, persistence, and severity of the allergic symptoms. Research indicates that patients suffering from allergic multimorbidity generally necessitate extensive medications and incur higher healthcare expenditures compared with those with a singular‐allergic condition.^7^

A study assessed the daily multitemporal incidence of allergic diseases utilizing an application program and analysis revealed a specific group (“extreme” allergy phenotype) combined rhinitis “High” (visual analogue scale >50/100) patterns—asthma “High”—conjunctivitis “High”, greatly impacted disease control and work efficiency.^2^ Additionally, findings from the French General Population Epidemiological Study, suggest that individuals with AS and AR had more severe symptoms, which were more likely to require combined pharmacological treatments for rhinitis, such as intranasal corticosteroids and oral antihistamines, than patients with AR alone.^8^, ^9^, ^10^ Consequently, when different allergic diseases coexist, diagnosis and treatment should be understood within the framework of multimorbidity, posing higher demands for a more integrated approach to the diagnosis, treatment, and management of these conditions throughout their course.

Allergens, as a causative factor of AR, stimulate the body to produce abnormal immune responses, ultimately leading to the onset of the disease. The duration and dosage of allergen exposure exhibited a positive correlation with both the type and severity of allergic diseases. A study conducted in Japan observed a significant positive correlation between the prevalence of AR in children and the concentration of pollen in the air.^11^ Interestingly, different concentrations of allergens affect different phenotypes of allergic diseases differently. For instance, patients allergic to grass pollen experience nasal and ocular sensitization when exposed to low doses of pollen, but the development of AS or wheezing symptoms requires higher concentrations of pollen exposure.^11^ Therefore, clarifying the distribution of allergens in AR multimorbidity and their interrelationships is essential for a comprehensive strategy for AR multimorbidity. The risk and severity of allergic diseases are influenced by allergen sensitization status, with monosensitization and polysensitization to various allergens manifesting distinct clinical outcomes. Polysensitization is associated with stronger overall immunoglobulin E (IgE) responses, more complex disease phenotypes, and more severe symptoms than monosensitization. Furthermore, individuals with polysensitization exhibited a higher risk of AS and AR, and a significant correlation was noted between the degree of allergic sensitization and the severity indicators of AS and AR.^12^, ^13^, ^14^, ^15^

To this end, the association between AR multimorbidity and allergen sensitization was initially investigated using a real‐world approach to understand AR multimorbidity from a novel perspective, furnish data for more detailed clinical investigations and pathogenesis studies, and potentially, guide the formulation of holistic management strategies for AR multimorbidity.

## 2 METHODOLOGY

### 2.1 Study population

**FIGURE 1:**
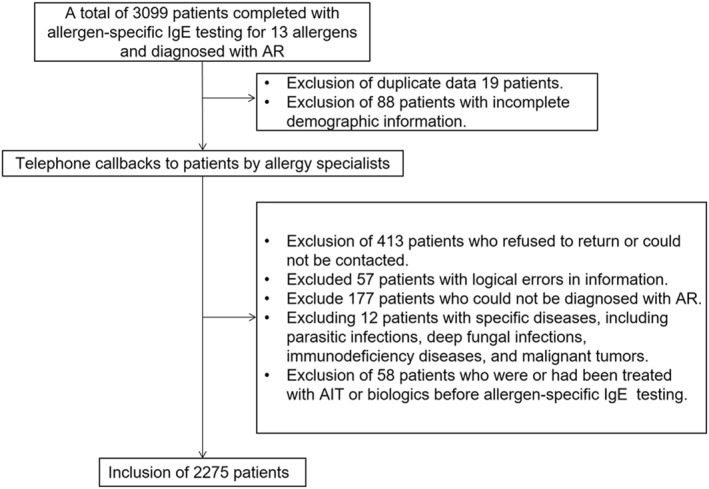
The flowchart of study participants. AIT, allergen immunotherapy.

This study reviewed patients who underwent serum allergen‐specific IgE testing for 13 common allergens and were initially diagnosed with AR by clinical specialists in the Third Affiliated Hospital of Sun Yat‐sen University from July 2021 to June 2023 (*n* = 3099). Exclusion criteria for this study were (1) patients with duplicate data (*n* = 19); (2) patients with incomplete demographic information (*n* = 88); (3) patients who refused to return or could not be contacted (*n* = 413); (4) patients with logically incorrect information (*n* = 57); (5) patients with an unconfirmed diagnosis of AR (*n* = 177); (6) patients with specific disease, including parasitic infections, deep fungal infections, immunodeficiency diseases, and malignant tumors (*n* = 12); and (7) patients who were being or had been treated with allergen immunotherapy (AIT) or biologics before allergen‐specific IgE testing (*n* = 58). Ultimately, 2275 patients were included in this analysis (Figure 1). This study was conducted in accordance with the protocol approved by the Ethics Committee of the Third Affiliated Hospital of Sun Yat‐sen University (EY2022‐142).

### 2.2 Allergen sensitization testing

A 3 mL sample of fresh peripheral venous blood was collected from each patient, and the serum was separated and stored at 4°C until further examination. An allergy screening system (Mediwiss Analytic GmbH, Moers, Germany) was used to determine allergen‐specific IgE of 13 common allergens, including 7 inhalant allergens of dust mites, cat dander, dog dander, cockroach, mold mixtures (Penicillium nodosum/Branched Sporothrix/Aspergillus fumigatus/Fungi sphaericus), tree pollen combinations (elm/willow/poplar), grass pollen combination (ragweed/artemisia argy/humulus/chenopodium), and 6 ingested allergens of milk, egg white, shrimp, crab, peanut, and soybean. Serum allergen‐specific IgE levels were expressed in international units (IU/mL), which were graded from level 0 to level 6: level 0 (0–0.34 IU/mL), level 1 (0.35–0.69 IU/mL), level 2 (0.70–3.49 IU/mL), level 3 (3.5–17.49 IU/mL), level 4 (17.5–49.9 IU/mL), and level 5 (50.0–100 IU/mL), level 6 (>100 IU/mL). An allergen‐specific IgE level measurement of 0.35 IU/mL or higher was defined as a positive result. For patients who underwent multiple allergen‐specific IgE tests, only the results of the first test were included.

### 2.3 Clinical data

Patients diagnosed with AR were selected, and their gender, age, ethnicity, origin, residence, and marriage were recorded and analyzed as demographic characteristics. Subsequently, the patients were called back by telephone in batches to find out whether they had other allergic diseases and whether there was a family history of allergic diseases in first‐ and second‐degree relatives.

### 2.4 Telephone follow‐up

Patients eligible for enrollment were interviewed by telephone by clinical physicians with expertise in allergy to assess the diagnosis of the following four allergic conditions in addition to AR: AS/cough variant asthma (CVA), AD/eczema, allergic conjunctivitis (AC), and food allergy (FA). Disease diagnoses were based on both physician diagnoses and self‐reports from patients, using a standardized interview questionnaire, designed based on the International Study of AS and Allergies in Childhood (ISAAC) questionnaire,^16^ the EuroPrevall Allergy Questionnaire,^17^ and published self‐report questionnaires on allergic diseases to assess the prevalence of allergic diseases among patients.^18^, ^19^ Informed consent was obtained verbally from the patients or their legal guardians before initiating the interview.

### 2.5 Statistical analysis

Descriptive data were described as median (25th, 75th percentile), while categorical data were described as N (%). The Wilcoxon rank‐sum test and the chi‐square test were used for comparisons between categorical groups. Multivariate logistic regression was applied to assess the association between allergen‐specific IgE and AR multimorbidity, adjusting for age, occupation, and family history of allergic diseases. Note that in the multivariate logistic regression analyses between allergen polysensitization and different phenotypes of AR multimorbidity, whenever there was a certain allergic multimorbidity, the case was classified in this phenotype. Therefore, there was a duplicate count when classifying different phenotypes of AR multimorbidity. Statistical analyses and visualizations were performed using R‐3.6.2, with all results considered statistically significant at *p <* 0.05.

## 3 RESULTS

### 3.1 Population characteristics

**FIGURE 2:**
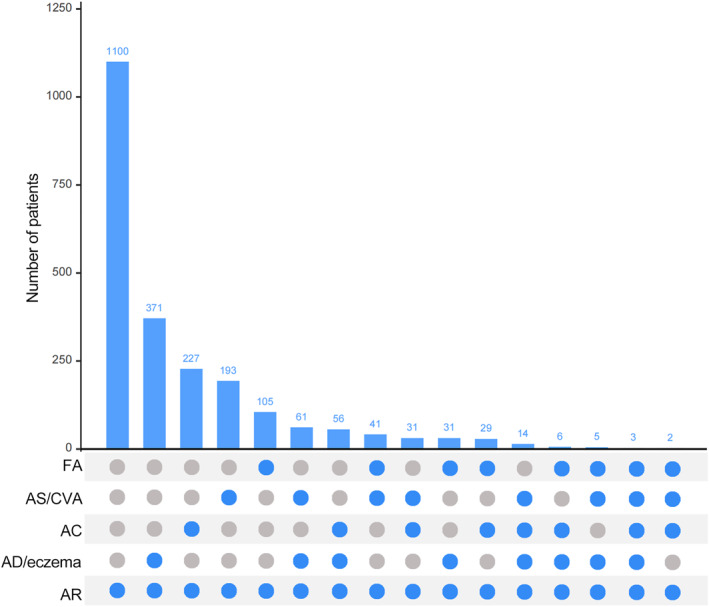
Prevalence of Allergic diseases. This diagram delineates the distribution of patients across various allergic conditions. The bar graph indicates the number of patients with different allergic multimorbidities, and the labeled solid scatter indicates different allergic conditions. AC, allergic conjunctivitis; AD, atopic dermatitis; AR, allergic rhinitis; AS, asthma; CVA, cough variant asthma; FA, food allergy.

Demographic data of the patients are summarized in Table 1. A total of 2275 patients diagnosed with AR were included in the analysis, with a median age of 18.0 (9.0, 29.0). Respectively, 1100 (48.4%) had AR alone, and 1175 (51.6%) had AR multimorbidity (Table 1). The distribution of allergic multimorbidity is shown in Figure 2, including the number of patients with each multimorbidity.

**TABLE 1:** Characteristics of patients with allergic diseases.

Variable	Total (*N* = 2275)	AR alone (*N* = 1100)	AR multimorbidity (*N* = 1175)	*p* value
Age median (25th, 75th percentile), years	18.0 (9.0, 29.0)	20.0 (10.0, 30.0)	15.0 (8.0, 27.5)	**<0.001**
<18 years old, *N* (%)	1129 (49.6)	508 (46.2)	621 (52.9)	**0.001**
≥18 years old, *N* (%)	1146 (50.4)	592 (53.8)	554 (47.1)	
Sex				
Male/Female, *N* (%)	1241 (54.5)/1034 (45.5)	612 (55.6)/488 (44.4)	629 (53.5)/546 (46.5)	0.334
Marriage				
Unmarried/Married, *N* (%)	1601 (70.4)/674 (29.6)	743 (67.5)/357 (32.5)	858 (73.0)/317 (27.0)	**0.005**
Occupation				
Unemployed, *N* (%)	48 (2.1)	24 (2.2)	24 (2.0)	**0.004**
Employed, *N* (%)	958 (42.1)	502 (45.6)	456 (38.8)	
Students, *N* (%)	1269 (55.8)	574 (52.2)	695 (59.1)	
Family history of allergic disorders				
Yes/No, *N* (%)	1491 (65.5)/784 (34.5)	685 (62.3)/415 (37.7)	806 (68.6)/369 (31.4)	**0.002**
Positive allergens (25th, 75th percentile)	1.0 (1.0, 2.0)	1.0 (1.0, 1.0)	1.0 (1.0, 2.0)	**<0.001**
Inhaled allergens positive/negative, *N* (%)	2220 (97.6)/55 (2.4)	1075 (97.7)/25 (2.3)	1145 (97.4)/30 (2.6)	0.663
Food allergens positive/negative, *N* (%)	451 (19.8)/1824 (80.2)	126 (11.5)/974 (88.5)	325 (27.7)/850 (72.3)	**<0.001**

*Note*: Bolded values indicate statistically significant differences between the two groups, i.e. *p* < 0.05.

Abbreviations: AR, allergic rhinitis; SD, standard deviation.

### 3.2 Allergen distribution in patients with AR multimorbidity differs from AR alone

**FIGURE 3:**
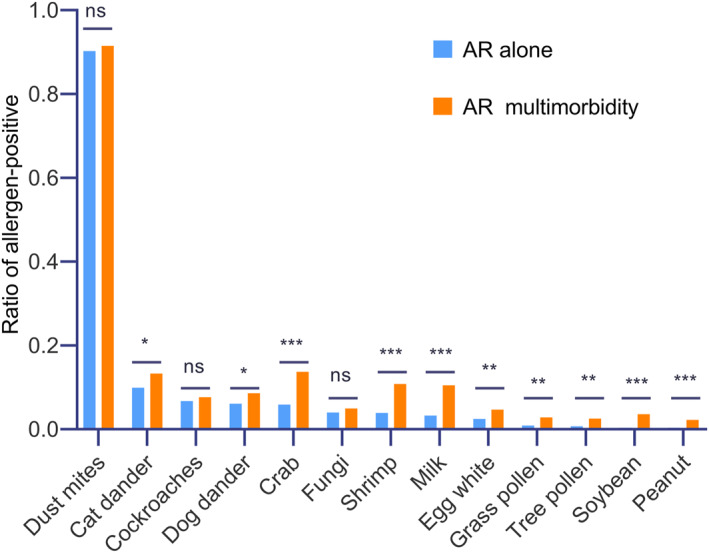
Ratio of allergen‐positivity. AR alone group: allergen‐positive cases/total number of AR alone (*n* = 1100). AR multimorbidity group: allergen‐positive cases/total number of AR multimorbidity (*n* = 1175). *p < 0.05, **p < 0.01, ***p < 0.001, and ns refer to non‐statistical significance. AR, allergic rhinitis.

**FIGURE 4:**
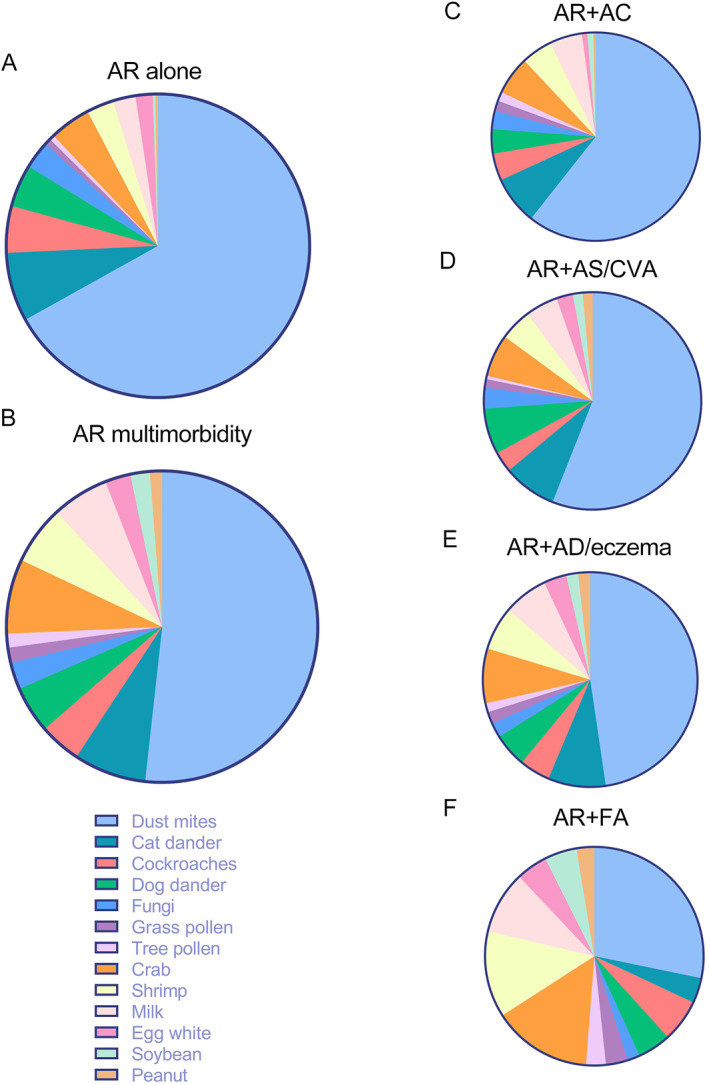
Patterns of allergen distribution. (A) Patterns of allergen distribution in AR alone. (B) Patterns of allergen distribution in AR multimorbidity. (C–F) Patterns of allergens for different AR multimorbidity phenotypes are shown separately. AC, allergic conjunctivitis; AD, atopic dermatitis; AR, allergic rhinitis; AS, asthma; CVA, cough variant asthma; FA, food allergy.

The results showed that positive allergens differed between AR alone and AR multimorbidity, with significant differences in peanut, soybean, egg white, milk, and shrimp (*p <* 0.001), tree pollen, and grass pollen (*p <* 0.01), and in crab, cat dander, and dog dander (*p <* 0.05) (Figure 3). Further analysis revealed that AR multimorbidity had a more diverse distribution pattern of positive allergens than AR alone and that the distribution pattern varied among different types of AR multimorbidity, especially in ingested allergens (Figure 4).

### 3.3 Association between the allergen polysensitization and different phenotypes of AR multimorbidity

A significant association was observed between the increasing number of positive allergen sensitization and AR multimorbidity, with an adjusted odds ratio (OR) of 1.64 (95% Confidence Interval [CI]: 1.49–1.83) (Table 2). Further stratified the association between the number of positive allergens and the risk of different phenotypes of AR multimorbidity. The adjusted ORs for increasing number of allergen positives: AR + AS/CVA (1.53 [95% CI 1.49–1.76]), AR + AD/eczema (1.82 [95% CI 1.59–2.09]), AR + AC (1.36 [95% CI 1.20–1.54]), and AR + FA (3.59 [95% CI 3.04–4.27]), indicating that the risk of AR coexist with AS/CVA, AD/eczema, AC, and FA increased by 0.5‐fold, 0.8‐fold, 0.3‐fold, and 2.5‐fold for each increasing number of positive allergens in the average (*p <* 0.001). Moreover, an increase in positive inhaled and ingested allergens was associated with the risk of AR multimorbidity (*P* trend <0.001). Interestingly, the increased number of positive ingested allergens had higher OR for AR multimorbidity compared to inhaled allergens, including AR + AS/CVA (1.58 vs. 1.57), AR + AD/eczema (2.06 vs. 1.68), AR + AC (1.49 vs. 1.31), and AR + FA (6.25 vs. 3.59) (*P* trend <0.001) (Table 2).

**TABLE 2:** Multivariate logistic analysis of association between allergen polysensitization and different phenotypes of AR multimorbidity.

Types of allergy diseases	Total		Inhaled allergens		Ingested allergens	
	OR (95% CI)	*p* value	OR (95% CI)	*p* value	OR (95% CI)	*p* value
AR versus AR multimorbidity	1.64 (1.49, 1.83)	**< 0.001**	1.46 (1.26, 1.68)	**< 0.001**	1.96 (1.69, 2.28)	**< 0.001**
AR versus AR + AC	1.36 (1.20, 1.54)	**< 0.001**	1.31 (1.09, 1.56)	**0.003**	1.49 (1.24, 1.79)	**< 0.001**
AR versus AR + AS/CVA	1.53 (1.34, 1.76)	**< 0.001**	1.57 (1.29, 1.92)	**< 0.001**	1.58 (1.30, 1.92)	**< 0.001**
AR versus AR + AD/eczema	1.82 (1.59, 2.09)	**< 0.001**	1.68 (1.39, 2.04)	**< 0.001**	2.06 (1.72, 2.49)	**< 0.001**
AR versus AR + FA	3.59 (3.04, 4.27)	**< 0.001**	2.00 (1.63, 2.46)	**< 0.001**	6.25 (5.02, 7.89)	**< 0.001**

*Note*: Bolded values indicate statistically significant differences between the two groups, i.e. *p* < 0.05.

Abbreviations: AC, allergic conjunctivitis; AD, atopic dermatitis; AR, allergic rhinitis; AS, asthma; CVA, cough variant asthma; FA, food allergy; OR, odds ratio.

### 3.4 Association between allergen‐specific IgE and the risk of AR multimorbidity

Subsequently, we investigated the association between allergen sensitization and the rate of occurrence of allergic multimorbidity. We analyzed the model with allergen sensitization and allergen‐specific IgE levels as the variables and the occurrence of allergic multimorbidity as the outcome (Tables 3 and 4). AR multimorbidity was significantly associated with the allergens dust mites, cat dander, grass pollen, soybean, and milk positivity, and it is noteworthy that dog dander was only observed to be associated with AR multimorbidity in the children group (*p <* 0.05) (Table 3). In the overall AR population, the risk of AR multimorbidity was significantly associated with the allergen‐specific IgE levels of dust mites, tree pollen, egg white, soybean, and milk (*p <* 0.05), with an average increase in the risk of 0.2‐fold, 1.5‐fold, 0.5‐fold, 6.1‐fold, and 1.3‐fold for each incremental grade in sensitization level, respectively. Specifically, in the pediatric patients, the risk of AR multimorbidity was associated with the allergen‐specific IgE levels of dust mites, cat dander, dog dander, soybean, milk, and shrimp (*p <* 0.05). Conversely, in adults, the risk of AR multimorbidity was associated with the allergen‐specific IgE levels of dust mites, tree pollen, soybean, and milk (*p <* 0.05) (Table 4).

**TABLE 3:** Multivariate logistic analysis of the association between allergen positivity and AR multimorbidity.

Positive allergens	Total		Adults group (≥18 years)		Children group (<18 years)	
	OR (95% CI)^a^	*p* value	OR (95% CI)^a^	*p* value	OR (95% CI)^a^	*p* value
Dust mites: Positive versus negative	1.90 (1.35, 2.66)	**<0.001**	2.01 (1.26, 3.21)	**0.003**	2.16 (1.26, 3.68)	**0.005**
Cockroaches: Positive versus negative	0.96 (0.67, 1.39)	0.845	0.84 (0.53, 1.33)	0.464	1.29 (0.68, 2.45)	0.428
Cat dander: Positive versus negative	1.58 (1.19, 2.10)	**0.002**	1.64 (1.11, 2.41)	**0.013**	1.63 (1.05, 2.52)	**0.029**
Dog dander: Positive versus negative	1.25 (0.88, 1.78)	0.209	0.96 (0.60, 1.54)	0.870	1.81 (1.03, 3.18)	**0.039**
Fungi: Positive versus negative	1.20 (0.78, 1.85)	0.401	1.30 (0.65, 2.59)	0.465	1.22 (0.69, 2.15)	0.491
Tree pollen: Positive versus negative	2.36 (0.92, 6.03)	0.073	2.68 (0.84, 8.6)	0.097	1.74 (0.33, 9.10)	0.514
Grass pollen: Positive versus negative	2.42 (1.02, 5.76)	**0.045**	3.03 (1.08, 8.46)	**0.034**	1.76 (0.36, 8.73)	0.487
Egg white: Positive versus negative	1.51 (0.88, 2.57)	0.134	2.01 (0.39, 10.38)	0.405	1.34 (0.75, 2.40)	0.318
Peanut: Positive versus negative	0.43 (0.08, 2.45)	0.342	0.33 (0.02, 5.47)	0.442	0.51 (0.05, 5.52)	0.576
Soybean: Positive versus negative	12.05 (2.73, 53.12)	**0.001**	13.06 (1.18, 144.94)	**0.036**	9.80 (1.39, 68.94)	**0.022**
Milk: Positive versus negative	3.49 (2.33, 5.24)	**<0.001**	3.71 (1.51, 9.13)	**0.004**	3.35 (2.11, 5.33)	**<0.001**
Crab: Positive versus negative	1.67 (0.97, 2.87)	0.064	2.05 (1.10, 3.80)	**0.024**	0.92 (0.29, 2.91)	0.883
Shrimp: Positive versus negative	1.85 (0.98, 3.46)	0.057	1.63 (0.73, 3.63)	0.232	3.18 (0.92, 11.02)	0.068

*Note*: Bolded values indicate statistically significant differences between the two groups, i.e. *p* < 0.05.

Abbreviations: AR, allergic rhinitis; CI, Confidence Interval; OR, odds ratio.

^a^ ORs for each allergen were multifactorial and adjusted for age, marriage, occupation, family history of allergic disorders, and whether other allergens sensitization or not.

**TABLE 4:** Multivariate logistic analysis of the association between allergen‐specific IgE levels and AR multimorbidity.

Allergen‐specific IgE levels^a^	Total		Adults group (≥18 years)		Children group (<18 years)	
	OR (95% CI)^b^	*p* trend	OR (95% CI)^b^	*p* trend	OR (95% CI)^b^	*p* trend
Dust mites	1.21 (1.14, 1.28)	**<0.001**	1.13 (1.05, 1.22)	**0.002**	1.31 (1.21, 1.42)	**<0.001**
Cockroaches	0.98 (0.81, 1.18)	0.819	0.95 (0.77, 1.18)	0.646	1.00 (0.67, 1.50)	0.993
Cat dander	1.09 (0.99, 1.21)	0.078	1.05 (0.93, 1.20)	0.406	1.19 (1.00, 1.42)	**0.044**
Dog dander	1.13 (0.94, 1.35)	0.183	1.02 (0.82, 1.27)	0.846	1.48 (1.03, 2.11)	**0.032**
Fungi	1.19 (0.94, 1.51)	0.156	1.14 (0.76, 1.71)	0.527	1.28 (0.94, 1.75)	0.111
Tree pollen	2.52 (1.18, 5.38)	**0.017**	2.54 (1.01, 6.41)	**0.048**	1.57 (0.35, 7.05)	0.555
Grass pollen	1.34 (0.85, 2.12)	0.205	1.33 (0.82, 2.17)	0.243	1.96 (0.58, 6.67)	0.280
Egg white	1.55 (1.07, 2.24)	**0.020**	1.50 (0.54, 4.18)	0.443	1.49 (0.99, 2.25)	0.056
Peanut	0.63 (0.18, 2.22)	0.478	0.74 (0.13, 4.20)	0.733	0.53 (0.07, 4.15)	0.548
Soybean	7.18 (2.05, 25.11)	**0.002**	7.38 (0.91, 59.93)	0.061	6.71 (1.14, 39.64)	**0.036**
Milk	2.32 (1.75, 3.06)	**<0.001**	1.71 (1.04, 2.83)	0.035	2.58 (1.83, 3.62)	**<0.001**
Crab	1.12 (0.84, 1.50)	0.442	1.37 (0.96, 1.95)	0.079	0.70 (0.39, 1.26)	0.240
Shrimp	1.37 (0.98, 1.90)	0.064	1.43 (0.88, 2.32)	0.153	2.09 (1.12, 3.90)	**0.020**

*Note*: Bolded values indicate statistically significant differences between the two groups, i.e. *p* < 0.05.

Abbreviations: AR, allergic rhinitis; CI, Confidence Interval; IgE, immunoglobulin E; OR, odds ratio.

^a^ Allergen‐specific IgE levels are classified as levels 0–6.

^b^ OR for each allergen were multifactorial and adjusted for age, marriage, occupation, family history of allergic disorder, and allergen‐specific IgE levels for all other allergens.

### 3.5 Association between allergen‐specific IgE and the risk of AR multimorbidity in different subgroups of children

Furthermore, we analyzed the association between the pattern of allergen sensitization and AR multimorbidity in different age subgroups of children. Sensitization to dust mites was significantly associated with AR multimorbidity in children aged 6–12 years old, and serum dust mite‐specific IgE level was significantly associated with AR multimorbidity in all age subgroups (*p* < 0.05). Additionally, sensitization to milk was significantly associated with AR multimorbidity in all age subgroups, and milk‐specific IgE level was significantly associated with AR multimorbidity in children aged >6 years old (*p* < 0.05) (Tables S1 and S2).

## 4 DISCUSSION

This is a real‐world study that retrospectively analyzed the association between allergen sensitization and AR multimorbidity and revealed that the distinctive pattern of allergen sensitization affects the risk of AR multimorbidity, including allergen positivity, allergen allergen‐specific IgE levels, and allergen polysensitization on the disease phenotype of AR multimorbidity. The present results provide a clinical data reference of allergen sensitization for AR in the Southern China population. These insights are poised to enhance future clinical investigations, validation and follow‐up studies, and the etiological pathogenesis of AR multimorbidity.

It is generally accepted that sensitization to different allergens causes associated allergic diseases, with inhaled allergens associated with airway allergic diseases and ingested allergens associated with AD and FA.^20^, ^21^, ^22^, ^23^, ^24^, ^25^, ^26^ However, there are now emerging findings that AS and AR were correlated with sensitization to ingested allergens.^27^ Our previous findings also showed that the dust mites had the highest proportion of patients with AD/eczema.^22^

It is evident that the association between allergen sensitization and allergic disease phenotypes is complex and remains to be explored, especially in the presence of allergic multimorbidity. We found that AR multimorbidity has a more diverse distribution pattern of positive allergens than AR alone. Moreover, the major allergen composition varies across allergic diseases, suggesting that the combined sensitizing effects of allergens are intertwined and may play an essential role in the onset and development of allergic multimorbidity.

In this investigation, we developed models to assess the risk associated with allergen polysensitization and the development of different AR multimorbidity phenotypes. Our analysis revealed that the risk of AR multimorbidity rose as the number of positive allergens increased (ORs: 1.64; 95%CI, 1.49–1.83). These results seem to be consistent with other research. A cross‐sectional case‐control study in Finnish adults found that the pathogenesis of sensitization to a single allergen was different from that of sensitization to multiple allergen types.^28^ Another Prediction of Allergies in Taiwanese Children (PATCH) study showed that the presence of combined sensitization to ingested and inhaled allergens in children aged 0–4 years increased the risk of developing allergic respiratory disease in early childhood.^29^ Polysensitization seems associated with allergic multimorbidity than monosensitization.^30^, ^31^

Interestingly, it seemed that AR patients presented with an increased number of ingested allergen sensitization rather than an increased number of inhaled allergen sensitization leading to a higher risk of allergic multimorbidity (inhaled allergen: OR 1.46, ingested allergen: OR 1.96). Similar results could be observed in AR + AC (inhaled allergens: OR 1.31, ingested allergens: OR 1.49) and AR + AD (inhaled allergens: OR 1.68, ingested allergens: OR 2.06). This phenomenon may be attributable to the impairment of skin barrier damage. According to the allergen dual exposure hypothesis, various types of environmental allergens, including food allergens, readily cross the compromised barrier when the skin barrier function is defective.^32^ Therefore, ingested allergens in patients with allergic multimorbidity should not be overlooked in clinical practice, as opposed to the more cautious screening of multiple allergen‐positive patients for underlying allergic multimorbidity, which can be diagnosed and intervened in as early a stage as possible, even though the patient's multisystemic allergy symptoms are not obvious.

To more precisely delineate which allergen sensitization may lead to the development of AR multimorbidity, and to ascertain the influence of allergen‐specific IgE levels, a prediction model was constructed. The results in Table 1 show that AR multimorbidity is more common in children; thus, the risk modeling was explored separately for adults and children. Additionally, we further stratified into the age groups 0–6 years, 6–12 years, and 12–18 years in children. It has also been shown that the distribution of multimorbidity varies across ages, with allergic multimorbidity being more common in children than adults in the prior study.^33^, ^34^ The risk of AR multimorbidity in both adults and children was associated with sensitization to dust mites and cat dander (*p <* 0.05), and increased with elevated dust mite‐specific IgE level (*P* trend <0.01). Increased risk of AR multimorbidity with elevated levels of cat and dog dander‐specific IgE was only observed in the children (*P* trend <0.05), which may be because the proportion of children was twice polysensitized to furry animal components as monosensitized, whereas adults are mainly monosensitized to furry animals.^35^, ^36^ However, AR multimorbidity only remained significantly associated with dust mite‐specific IgE but disappeared in association with cat and dog dander in association with different minor subgroups. This situation may be attributed to the small sample size. Our study is consistent with those of some previous studies that dust mites and cat dander, as perennial allergens with prolonged exposure, were associated with multimorbidity, especially in children. These long‐lived allergens may play an important role in multimorbidity.^33^, ^37^ Dust mites cause allergies mainly from their excreta (feces) and shed skin shells which enter the airways while breathing, thus causing respiratory allergic reactions. Since the cysteine proteases in dust mites have a disruptive effect on the skin barrier function, which is closely associated with exacerbation of exogenous AD, it can also stimulate protease‐activated receptors and trigger cytokine release to drive the development of skin inflammation.^38^, ^39^

In addition, it has been found that there may be cross‐reactivity with food allergens, called dust mite‐food allergy syndrome (DMFAS). For example, the dust mite component *Der p*10, which is a pro‐myosin, is also a major shrimp and crab allergen.^40^ Moreover, dust mites found in human intestinal mucosa can lead to localized inflammation and increase the permeability of the intestinal epithelium, resulting in the development of gastrointestinal‐allergic symptoms.^38^, ^39^ Overall, sensitization to dust mites can initiate a complex immune response, potentially culminating in a spectrum of allergic diseases.

We found that among the ingested allergens, milk and soybean were associated with an increased risk of AR multimorbidity. This may be related to the previously mentioned skin barrier damage. It was also found that allergen‐specific IgE levels in milk and soybean were associated with an increased risk of AR multimorbidity in children. Studies have shown that AD patients are prone to inflammation after ingesting specific food allergens.^41^, ^42^, ^43^ FA is present in about one‐third of children with moderate‐to‐severe AD, with milk, eggs, soybeans, and peanuts being the most commonly associated allergens in young children, while in older children or adults, allergies to pollen‐related foods, including apples, carrots, celery, and hazelnuts, are more common triggers.^44^, ^45^, ^46^ The present study provides preliminary insights into the impact of specific ingested allergens on AR multimorbidity, and attention should be paid to the importance of milk in the different phenotypes of AR multimorbidity, but these results need to be interpreted with caution.

This study has several limitations. Initially, while the allergen sensitization patterns yield insights pertinent to a specific dimensional area, the generalizability of these findings is limited due to the single‐center nature of the study, which was conducted in Guangzhou, China. Consequently, further multi‐center clinical validation of these findings is necessary. Secondly, the study employed a semi‐quantitative allergen‐specific IgE assay using 0.35 IU/mL as the threshold for judgment, which can be affected by age and allergen type and may be inaccurate in identifying non‐IgE‐mediated or mixed‐mediated allergic diseases. Additionally, while we diagnosed FA through a comprehensive evaluation of clinical questionnaires, typical symptoms, and IgE testing, the absence of oral food challenge test and skin prick test necessitates careful interpretation of these diagnoses. Ultimately, this study did not collect information on AR severity or patients' responses to standard treatments. This limitation may impact the applicability of our findings to patients with varying clinical characteristics. Further studies with a larger sample size and a prospective design are needed to address these gaps.

### 4.1 Implications

The results of this study highlight the association between the sensitization patterns of serum allergen‐specific IgE and AR multimorbidity, suggesting that the allergen sensitization patterns in patients with AR multimorbidity differ from AR alone, offering critical insights for the clinical management of AR multimorbidity. These findings underscore the importance of assessing sensitization to ingested allergens in patients with AR multimorbidity, even in the absence of gastrointestinal symptoms. Correspondingly, in patients who exhibit sensitivity to multiple allergens, attention should be paid to the potential risk of concomitant allergic multimorbidity, even if they currently display symptoms of AR alone.

## 5 CONCLUSION

AR multimorbidity has a unique allergen sensitization pattern and a more abundant positive allergen profile than AR alone. The risk of AR multimorbidity increases with the number of positive allergens, indicating that the ORs of developing different phenotypes of AR multimorbidity are associated with polysensitization. Furthermore, the risk of AR multimorbidity is higher with an increasing number of ingested allergens compared with inhaled allergens, regardless of AR multimorbidity phenotype.

## AUTHOR CONTRIBUTIONS

**Ya‐Ting Li**: Writing—original draft; data curation; visualization; formal analysis. **Qi‐Qing Ye**: Data curation; visualization; formal analysis; writing—original draft. **Ya‐Xin Lu**: Data curation; formal analysis; writing—original draft; visualization. **Ke‐Xin Yang**: Investigation. **Ping‐Ping Zhang**: Investigation. **Chang Chen**: Writing—review and editing. **Min Zhou**: Conceptualization; writing—review and editing; methodology. **Pei‐Ying Feng**: Conceptualization; writing—review and editing; methodology. **Zhuang‐Gui Chen**: Conceptualization; writing—review and editing; methodology; project administration; supervision; resources.

## CONFLICT OF INTEREST STATEMENT

The researchers declare that the study was carried out without any business or monetary affiliations that could be interpreted as a possible conflict of interests.

## Supporting information

Supporting Information S1

## Data Availability

The data that support the findings of this study are available on request from the corresponding author. The data are not publicly available due to privacy or ethical restrictions.
