# Supplementary material for: Allergen sensitization patterns: Allergic rhinitis with multimorbidity versus alone—A real‐world study
Source: Clin Transl Allergy. 2025 Jan 22;15(1):e70030. doi: 10.1002/clt2.70030 (PMC11753898; doi:10.1002/clt2.70030)
Supplement: Supplementary file 1 — Supporting Information S1 [file CLT2-15-e70030-s001.doc]

**Table S1.** **Multivariate logistic analysis of association between allergen sensitization and AR multimorbidity in different subgroups of children.**

| **Positive allergens** | **≤ 6 years** **(n=122)** | |  | **6-12 years (n=612)** | |  | **>12 years (n=337)** | |
| --- | --- | --- | --- | --- | --- | --- | --- | --- |
|  | **OR (95%CI) ^a^** | ***P* value** |  | **OR (95%CI) ^a^** | ***P* value** |  | **OR (95%CI) ^a^** | ***P* value** |
| Dust mites: positive vs. negative | 2.04 (0.51，8.10) | 0.312 |  | 3.25 (1.55，6.80) | **0.002** |  | 0.30 (0.05，1.82) | 0.192 |
| Cockroache: positive vs. negative | _^b^ | _^b^ |  | 1.66 (0.64，4.30) | 0.294 |  | 0.75 (0.27，2.09) | 0.579 |
| Cat dander: positive vs. negative | 5.04 (0.52，49.16) | 0.164 |  | 1.78 (0.95，3.32) | 0.071 |  | 1.27 (0.60，2.71) | 0.533 |
| Dog dander: positive vs. negative | _^b^ | _^b^ |  | 1.26 (0.61，2.61) | 0.533 |  | 2.37 (0.73，7.66) | 0.150 |
| Fungi: positive vs. negative | 2.41 (0.23，24.95) | 0.461 |  | 1.27 (0.55，2.91) | 0.571 |  | 0.82 (0.30，2.28) | 0.706 |
| Tree pollen: positive vs. negative | _^b^ | _^b^ |  | 1.83 (0.29，11.72) | 0.522 |  | _^b^ | _^b^ |
| Grass pollen: positive vs. negative | _^b^ | _^b^ |  | 0.56 (0.07，4.41) | 0.580 |  | _^b^ | _^b^ |
| Egg white: positive vs. negative | 1.62 (0.48，5.52) | 0.438 |  | 1.18 (0.52，2.70) | 0.693 |  | 0.41 (0.07，2.38) | .319 |
| Peanut: positive vs. negative | 0.23 (0.01，5.42) | 0.363 |  | _^b^ | _^b^ |  | _^b^ | _^b^ |
| Soybean: positive vs. negative | 3.70 (0.32，42.07) | 0.292 |  | _^b^ | _^b^ |  | _^b^ | _^b^ |
| Milk: positive vs. negative | 2.27 (0.60，8.66) | **0.023** |  | 3.97 (2.09，7.54) | **< 0.001** |  | 4.30 (1.50，12.33) | **0.007** |
| Crab: positive vs. negative | _^b^ | _^b^ |  | 1.10 (0.16，7.75) | 0.926 |  | 1.19 (0.46，6.35) | 0.960 |
| Shrimp: positive vs. negative | _^b^ | _^b^ |  | 1.97 (0.25，15.49) | 0.519 |  | 2.51 (0.40，15.77) | 0.409 |

^a^ OR for each allergen were multifactorial and adjusted for age, marriage, occupation, family history of allergic disorders, and whether other allergens sensitization or not. ^b^ There were too few patients to perform statistical analyses. Abbreviations: AR, allergic rhinitis; CI, Confidence Interval; OR, odds ratio.

**Table S2. Multivariate logistic analysis of association between allergen-specific IgE levels and AR multimorbidity in different subgroups of children.**

| **Allergen-specific IgE levels ^a^** | **≤ 6 years (n=122)** | |  | **6-12 years (n=612)** | |  | **>12 years (n=337)** | |
| --- | --- | --- | --- | --- | --- | --- | --- | --- |
|  | **OR (95%CI) ^b^** | ***P* trend** |  | **OR (95%CI) ^b^** | ***P* trend** |  | **OR (95%CI) ^b^** | ***P* trend** |
| Dust mites | 1.44 (1.09，1.91) | **0.011** |  | 1.35 (1.21，1.50) | **< 0.001** |  | 1.32 (1.13，1.55) | **< 0.001** |
| Cockroache | _^c^ | _^c^ |  | 1.14 (0.62，2.08) | 0.675 |  | 0.75 (0.39，1.42) | 0.371 |
| Cat dander | 3.31 (0.65，16.98) | 0.151 |  | 1.14 (0.91，1.44) | 0.262 |  | 1.15 (0.85，1.54) | 0.363 |
| Dog dander | _^c^ | _^c^ |  | 1.22 (0.80，1.88) | 0.355 |  | 1.83 (0.85，3.95) | 0.125 |
| Fungi | 2.43 (0.25，23.46) | 0.444 |  | 1.45 (0.81，2.60) | 0.215 |  | 1.17 (0.71，1.91) | 0.543 |
| Tree pollen | _^c^ | _^c^ |  | 2.45 (0.39，15.44) | 0.341 |  | _^c^ | _^c^ |
| Grass pollen | _^c^ | _^c^ |  | 0.86 (0.18，4.21) | 0.855 |  | _^c^ | _^c^ |
| Egg white | 1.87 (0.65，5.38) | 0.244 |  | 1.23 (0.70，2.16) | 0.466 |  | 0.81 (0.22，3.05) | 0.761 |
| Peanut | 0.27 (0.02，4.29) | 0.352 |  | _^c^ | _^c^ |  | _^c^ | _^c^ |
| Soybean | 3.92 (0.51，30.19) | 0.189 |  | _^c^ | _^c^ |  | _^c^ | _^c^ |
| Milk | 2.83 (0.85，9.35) | 0.089 |  | 2.73 (1.72，4.34) | **< 0.001** |  | 2.58 (1.29，5.18) | **0.008** |
| Crab | 0.03 (0，619.81) | 0.482 |  | 2.05 (0.24，1.44) | 0.245 |  | 1.02 (0.46，2.26) | 0.960 |
| Shrimp | _^c^ | _^c^ |  | 2.28 (0.88，5.95) | 0.091 |  | 1.42 (0.62，3.27) | 0.409 |

^a^Allergen-specific IgE levels are classified as levels 0-6. ^b^ OR for each allergen were multifactorial and adjusted for age, marriage, occupation, family history of allergic disorder, and allergen-specific IgE levels for all other allergens. ^c^ There were too few patients to perform statistical analyses. Abbreviations: AR, allergic rhinitis; CI, Confidence Interval; IgE, immunoglobulin E; OR, odds ratio.
